# Micromagnetic Simulation of *L*1_0_-FePt-Based Exchange-Coupled-Composite-Bit-Patterned Media with Microwave-Assisted Magnetic Recording at Ultrahigh Areal Density

**DOI:** 10.3390/mi12101264

**Published:** 2021-10-17

**Authors:** Pirat Khunkitti, Naruemon Wannawong, Chavakon Jongjaihan, Apirat Siritaratiwat, Anan Kruesubthaworn, Arkom Kaewrawang

**Affiliations:** 1 Department of Electrical Engineering, Faculty of Engineering, Khon Kaen University, Khon Kaen 40002, Thailand; piratkh@kku.ac.th (P.K.); chavakon.j@kkumail.com (C.J.); apirat@kku.ac.th (A.S.); anankr@kku.ac.th (A.K.); 2 Seagate Technology (Thailand) Co., Ltd., Nakhon Ratchasima 30170, Thailand; yongyangbobby@gmail.com

**Keywords:** bit-patterned media, exchange-coupled-composite media, microwave-assisted magnetic recording, hysteresis loop

## Abstract

In this work, we propose exchange-coupled-composite-bit-patterned media (ECC-BPM) with microwave-assisted magnetic recording (MAMR) to improve the writability of the magnetic media at a 4 Tb/in^2^ recording density. The suitable values of the applied microwave field’s frequency and the exchange coupling between magnetic dots, *A*_dot_, of the proposed media were evaluated. It was found that the magnitude of the switching field, *H*_sw_, of the bilayer ECC-BPM is significantly lower than that of a conventional BPM. Additionally, using the MAMR enables further reduction of *H*_sw_ of the ECC-BPM. The suitable frequency of the applied microwave field for the proposed media is 5 GHz. The dependence of *A*_dot_ on the *H*_sw_ was additionally examined, showing that the *A*_dot_ of 0.14 pJ/m is the most suitable value for the proposed bilayer ECC-BPM. The physical explanation of the *H*_sw_ of the media under a variation of MAMR and *A*_dot_ was given. Hysteresis loops and the magnetic domain of the media were characterized to provide further details on the results. The lowest *H*_sw_ found in our proposed media is 12.2 kOe, achieved by the bilayer ECC-BPM with an *A*_dot_ of 0.14 pJ/m using a 5 GHz MAMR.

## 1. Introduction

Recently, the capacity of hard disk drives has been heading towards a stalemate since the thermal stability of the conventional media has reached its limitation [1,2,3,4,5,6]. In order to increase recording densities, several techniques have been proposed to overcome the stability limitation, such as exchange-coupled-composite (ECC) media, heat-assisted magnetic recording (HAMR) bit-patterned media (BPM), and microwave-assisted magnetic recording (MAMR) [1,7,8,9,10,11,12,13,14]. The ECC media have been introduced to improve the magnetic properties of the media; the major goal is to reduce the magnitude of the switching field, *H*_sw_ [7,12,13]. The ECC media consist of magnetically isolated layers, which are the soft and the hard magnetic layers. The interface exchange coupling between two layers can provide a lower *H*_sw_ than the conventional one. It therefore enables the use of smaller media grain sizes with higher magnetic anisotropy, *K*_u_, at high areal densities [7]. BPM technology has been extensively proposed to solve the magnetic transition noise and the interaction between magnetic bits of the conventional media since these factors could be the crucial issues at ultrahigh areal densities [1]. The principle of BPM is the magnetic separation of each magnetic bit, which eliminates the magnetic transition noise and yields a very low interaction between bits [1]. In previous research, the combination of ECC-BPM was reported, demonstrating that this combined technique can increasingly reduce the *H*_sw_ of the media beyond that using each technique individually [12]. Microwave-assisted magnetic recording (MAMR) is one promising technology for achieving higher areal densities [14,15,16,17,18]. The goal of this technique is to reduce the *H*_sw_ of the media by using an AC-magnetic field, *H*_ac_, operated at a microwave range. During the writing process of MAMR, the *H*_ac_ is applied to the media simultaneously with the writing field, *H*_dc._ This strategy can increasingly reduce the energy barrier of the media by the ferromagnetic resonance (FMR) phenomenon. As a result, switching the magnetization of the media becomes easier. A significant reduction in the *H*_sw_ of the media under the use of MAMR can be seen in several recent publications [14,15,16,17,18].

As it is generally known that the maximum write head field is limited, it is challenging to continuously reduce the *H*_sw_ to enable the use of higher *K*_u_ materials as a media at higher recording densities [19]. Therefore, we proposed a novel technique to improve the writability of the media by the combination of three technologies, including the ECC media, BPM, and MAMR. The proposed media was targeted for use at an areal density of 4 Tb/in^2^. The hybrid magnetic recording media based on the FePt alloy with the face-centered tetragonal *L*1_0_ structure, *L*1_0_-FePt, was focused. The object-oriented micromagnetic framework (OOMMF) software [20] was used in the simulations implementing the Landau–Lifshitz–Gilbert (LLG) equation. 

## 2. Modeling and Analytical Methodology

**Figure 1:**
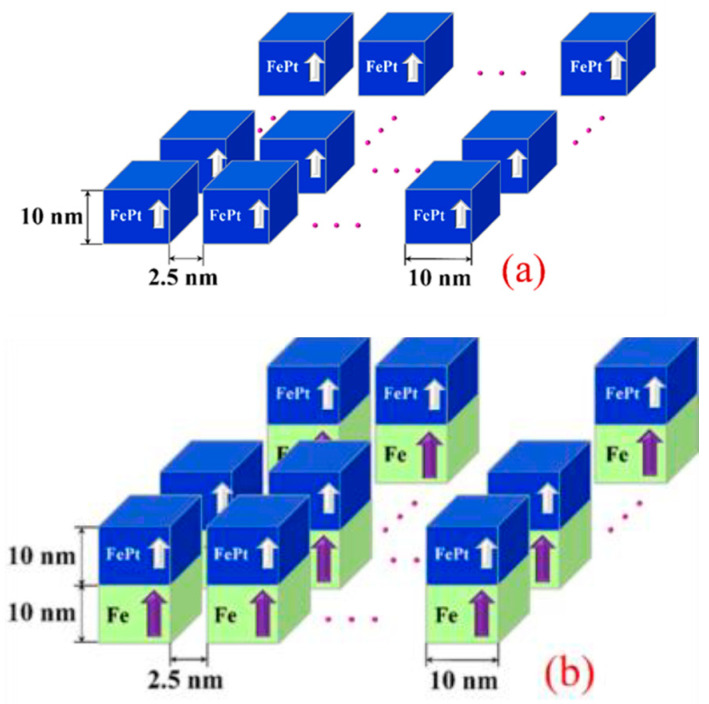
(**a**) Single layer BPM and (**b**) bilayer ECC-BPM.

The model structures of the *L*1_0_-FePt-based single-layer BPM and the *L*1_0_-FePt/Fe bilayer ECC-BPM for an areal density of 4 Tb/in^2^ are shown in Figure 1a,b, respectively. The media configuration has been determined on the basis of BPM and ECC-BPM requirements. To achieve an areal density of 4 Tb/in^2^, the cube dot size of single-layer BPM of 10 × 10 × 10 nm^3^ and spacing between dots of 2.5 nm were assumed [12]. The bilayer ECC-BPM was introduced by magnetically adding a soft Fe layer with a 10 nm thickness under the FePt BPM. The Fe added layer was assumed to have the same dot pattern as the single-layer BPM. The magnetization of each layer was initially aligned along the +z direction for both media. The magnetic properties of the proposed media are detailed as follows: the *L*1_0_-FePt hard layer had a saturation magnetization, *M*_s_, of 1.175 MA/m, and a *K*_u_ of 2.8 MJ/m^3^. The Fe soft layer had *M*_s_ of 1.71 MA/m and *K*_u_ of 100 J/m^3^. For the bilayer media, the exchange coupling between soft and hard layers, *A*_ex_, was assumed to be 25 pJ/m, whereas the exchange coupling between magnetic dots, *A*_dot_, varied from 0.1 to 0.3 pJ/m. This variation of *A*_dot_ was based on the possible values of the filled material between the dot spacing, as can be seen in the literature [21,22]. In calculations, the time-varying magnetization was described by the LLG equation, given as Equation (1) [23]:

(1) $$\frac{\partial \vec{M}}{\partial t}=-\left|\gamma \right|\mu_{0}\left(\vec{M}\times {\vec{H}}_{eff}+\frac{\propto }{M_{s}}\vec{M}\times \left(\vec{M}\times {\vec{H}}_{eff}\right)\right)$$

where $\vec{M}$ is the magnetization, *γ* is the gyromagnetic ratio, and ${\vec{H}}_{eff}$ is the effective magnetic field given by Equation (2):

(2) $${\vec{H}}_{eff}={\vec{H}}_{dc}+{\vec{H}}_{ex}+{\vec{H}}_{de}+{\vec{H}}_{k}+h_{ac}\sin\left(2\pi f_{ac}t\right){\vec{a}}_{x}$$

where ${\vec{H}}_{dc},\;{\vec{H}}_{ex},\;{\vec{H}}_{de},\;{\vec{H}}_{k},\;h_{ac},\;f_{ac},$ and ${\vec{a}}_{x}$ are the external static magnetic field, the exchange field between nearest-neighbor cells, the demagnetizing field, the field due to uniaxial anisotropy, the amplitude of microwave field, the microwave frequency, and the unit vector along the x-axis, respectively. 

To investigate the writability of the media, the external DC write field, *H*_dc_, was applied to the media in the z-direction. The write head field region was defined as the region where the magnetization of the media can be written by the write head field. The *H*_sw_ of the media was collected in the condition that *M* = −0.8*M*_s_. For the MAMR included, the *H*_ac_ was simultaneously applied to *H*_dc_ with a magnitude of 100 mT in the x-direction. The *H*_sw_ of the proposed ECC-BPM was examined at various frequencies of *H*_ac_. Since *A*_dot_ typically has an influential impact on the *H*_sw_, the dependence of *A*_dot_ on the *H*_sw_ was also taken into account. Then, the hysteresis loops and magnetic domain of a bilayer ECC-BPM were determined and compared with those of a single-layer BPM.

## 3. Results and Discussions

**Figure 2:**
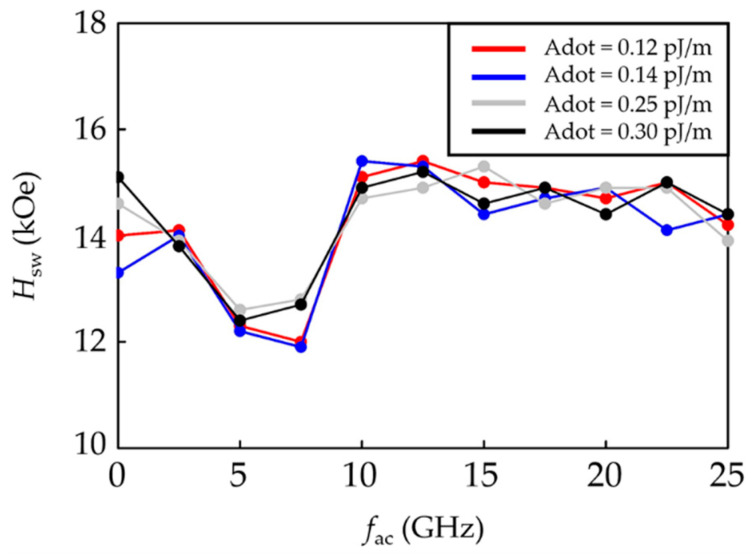
*H*_sw_ of the bilayer ECC-BPM versus *f*_ac_ at various *A*_dot_.

The *H*_sw_ of the proposed bilayer ECC-BPM at different *A*_dot_ values was investigated at *f*_ac_ between 0 and 25 GHz, as shown in Figure 2. It was discovered that changing *A*_dot_ or *f*_ac_ could change the *H*_sw_. Without MAMR, the media with an *A*_dot_ of 0.14 pJ/m have the lowest *H*_sw_, followed by those with an *A*_dot_ of 0.12, 0.25, and 0.30 pJ/m, respectively. A variation of *A*_dot_ under the MAMR indicates a similar trend of *H*_sw_ for all *f*_ac_. When the MAMR was performed, it revealed that increasing the *f*_ac_ from 0 to 2 GHz slightly changes the *H*_sw_ of the media. Then, the *H*_sw_ was dramatically reduced to the range of *f*_ac_ about 5–7.5 GHz. Above *f*_ac_ of 10 GHz, the *H*_sw_ of all media tended to have a higher *H*_sw_ than that without MAMR and was insignificantly changed with varying *f*_ac_. From the results, the media with *A*_dot_ = 0.25, 0.30 pJ/m indicate their lowest *H*_sw_ at a *f*_ac_ of 5 GHz, whereas the lowest *H*_sw_ of media with *A*_dot_ = 0.12, 0.14 pJ/m occurs at a *f*_ac_ of 7.5 GHz. The lowest *H*_sw_ found in this evaluation is 11.9 kOe, achieved by the media with an *A*_dot_ of 0.14 pJ/m with MAMR at a *f*_ac_ of 7.5 GHz. However, since the resonance frequency of our proposed system is 5 GHz, we therefore examined more details of *A*_dot_’s influence on the *H*_sw_ at this frequency.

**Figure 3:**
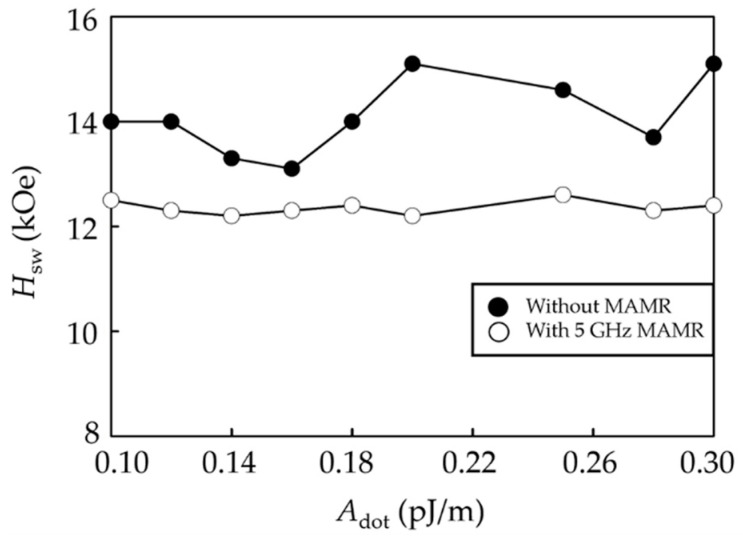
*H*_sw_ of the bilayer ECC-BPM with and without HAMR as a function of *A*_dot_.

Figure 3 displays the *H*_sw_ of the bilayer ECC-BPM as a function of *A*_dot_, in the cases without and with the MAMR at *f*_ac_ = 5 GHz. Overall, it shows that using MAMR can significantly reduce the *H*_sw_ of the media for all *A*_dot_ values. Additionally, the media with MAMR indicate less variation of *H*_sw_ with a varying *A*_dot_ than that without MAMR. The lowest *H*_sw_ of ECC-BPM without and with MAMR is 13.1 kOe at *A*_dot_ = 0.16 pJ/m and 12.2 kOe at *A*_dot_ = 0.14 pJ/m, respectively. The *H*_sw_ alternation with varying *A*_dot_ at other *f*_ac_ is supposed to provide a higher *H*_sw_ than that at *f*_ac_ = 5 GHz due to its larger conventional value and therefore is not considered in this work. 

In a case without MAMR, the media with higher *A*_dot_ tend to have higher *H*_sw_. A magnitude of *A*_dot_ normally represents an exchange interaction between magnetic dots of the media. This interaction indicates an interacting force between adjacent magnetizations. At higher *A*_dot_, each magnetic dot is strongly related to each other due to a massive exchange interaction between them. When the write head field is applied to the media, the magnetization of the media with higher *A*_dot_ is more homogeneously processed, then it is more difficult to be switched. 

The physical reason why a variation of *f*_ac_ can alter the *H*_sw_ of media with MAMR can be explained by the FMR phenomenon, as follows: when the magnetization precession of a ferromagnetic material is under the effect of an external magnetic field, the FMR frequency, *f*_0_, can be obtained by *f*_0_ = *γ*(*H −* 4π*M*_s_,_eff_) [24] where *H* is the magnitude of media switching field with an absence of MAMR and *M*_s,eff_ is the effective saturation magnetization of the bilayer ECC-BPM. The FMR itself can exert additional energy on the magnetization through the resonance phenomenon, which causes the additional magnetization precession. The magnitude of this added energy depends on the frequency of the external microwave field. The maximum added energy occurs when the *f*_ac_ is synchronized with the FMR frequency of the system, which typically provides a significant reduction in *H*_sw_. In addition, the reason why the media with MAMR have a lower *H*_sw_ than the conventional media is that the magnetization precession of the media subjected to the microwave field receives the additional energy exerted by the microwave field. The magnetization of the hard layer, therefore, requires lower *H*_sw_ for switching than in a case without MAMR. In particular, this additional torque energy becomes massive when the frequency of MAMR corresponds with the precession frequency of its FMR. 

**Figure 4:**
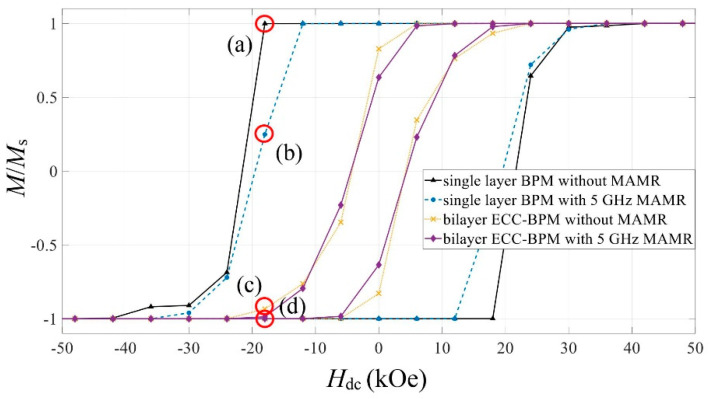
The hysteresis loops of the single-layer BPM and the bilayer ECC-BPM with and without MAMR (*A*_dot_ = 0.14 pJ/m for both media).

**Figure 5:**
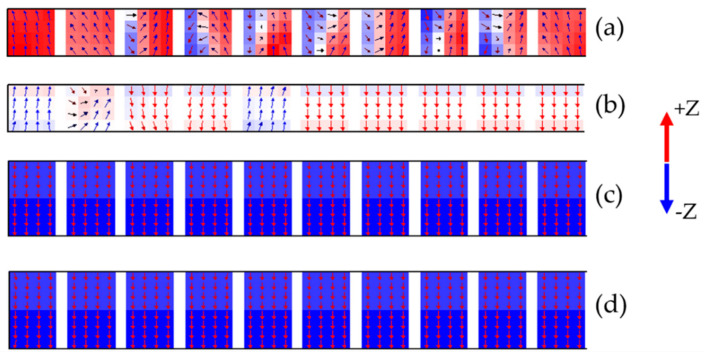
Magnetic domain from cross-sectional side view of (**a**) Single-layer BPM; (**b**) Single-layer BPM with MAMR; (**c**) Bilayer ECC-BPM; (**d**) Bilayer ECC-BPM with MAMR at *H*_dc_ = −18 kOe.

A comparison of the hysteresis loops of the single-layer BPM and the bilayer ECC-BPM for cases without and with 5 GHz MAMR is indicated in Figure 4. The *A*_dot_ of 0.14 pJ/m was selected for this characterization for both media since this value previously demonstrates the lowest *H*_sw_ under MAMR. In this figure, the *H*_sw_ is determined at the point that *M = −*0.8*M*_s_. From the results, the single-layer BPM has the *H*_sw_ of 27 kOe and 26 kOe for cases without and with MAMR, respectively. By adding the Fe soft layer to the single-layer BPM, the *H*_sw_ of the bilayer ECC-BPM for the cases without and with MAMR was reduced to 13.3 kOe and 12.2 kOe, respectively, which are significantly lower than that of the single-layer BPM. To provide more information about the magnetization orientation of the media, the magnetic domain of four media was characterized at the points where *H*_dc_ = −18 kOe since this particular *H*_dc_ can indicate the difference between them. Figure 5a,d show the magnetic domain from the cross-sectional side view of media at the points shown by circles (a–d) in Figure 4, respectively. It is seen that the magnetization of the single-layer BPM with MAMR contains more reversed magnetization than that without MAMR, demonstrating the effects of MAMR assisting the magnetization switching. Additionally, the magnetization of the bilayer ECC-BPMs is greatly reversed when compared to the single-layer BPM. The use of the MAMR in the bilayer ECC-BPM indicates a slightly better reversal of magnetization. 

From overall evaluations, the lowest *H*_sw_ found in this work is 12.2 kOe, achieved by the proposed bilayer ECC-BPM with an *A*_dot_ of 0.14 pJ/m using a 5 GHz MAMR, which is below the maximum write head field existed in the literature. Therefore, this proposed bilayer ECC-BPM could be another choice as the magnetic media for an areal density of 4 Tb/in^2^ of data storage technology. It should be noted that the suitable value of *A*_dot_ and *f*_ac_ for other media and areal densities recording systems needs to be carefully optimized. The theoretical findings can be the guidelines for its experimental verification as well as further development of magnetic media in the future. 

## 4. Conclusions

In this work, the *L*1_0_-FePt single-layer BPM and *L*1_0_-FePt/Fe bilayer ECC-BPM with MAMR technology were proposed to improve the writability of the magnetic media at a 4 Tb/in^2^ recording density. It was found that the *H*_sw_ bilayer ECC-BPM was significantly lower than that of the single-layer BPM. The *H*_sw_ of those media could be increasingly reduced by using MAMR. The suitable frequency of the applied microwave field was 5 GHz, which was consistent with the FMR of the system. The suitable value of *A*_dot_ for the proposed bilayer ECC-BPM under MAMR was 0.14 pJ/m. The physical explanation of the *H*_sw_ of the medium regarding a variation of MAMR and *A*_dot_ was given. The hysteresis loops and magnetic domain of the medium have been considered to provide further details on the simulation results. The lowest *H*_sw_ found in this proposed medium is 12.2 kOe, achieved by the bilayer ECC-BPM with an *A*_dot_ of 0.14 pJ/m using a 5 GHz MAMR. Findings can be used for the future development of ultra-high areal densities magnetic recording technology.
